# Cross-Sectional Analysis of Mental Health among University Students: Do Sex and Academic Level Matter?

**DOI:** 10.3390/ijerph191912670

**Published:** 2022-10-03

**Authors:** Carsten Müller, Kareem El-Ansari, Walid El Ansari

**Affiliations:** 1 Department of Applied Health Sciences, Hochschule für Gesundheit, 44801 Bochum, Germany; 2 University Sports, University of Münster, 48149 Münster, Germany; 3 Faculty of Medicine, Ain Shams University, Cairo 11591, Egypt; 4 Department of Surgery, Hamad General Hospital, Hamad Medical Corporation, Doha 3050, Qatar; 5 College of Medicine, Qatar University, Doha 2713, Qatar; 6 Weill Cornell Medicine—Qatar, Doha 24144, Qatar

**Keywords:** self-rated health, self-esteem, mental health, university students, stress, depression, anxiety, health psychology, academic achievement

## Abstract

University students’ mental health and well-being is a growing public health concern. There is a lack of studies assessing a broad range of mental health domains by sex and academic level of study. This cross-sectional online survey of BSc, MSc, and PhD students (*n* = 3353, 67% female) enrolled at one university in Germany assessed a wide scope of mental health domains, covering positive (i.e., self-rated health, self-esteem, student engagement) and negative aspects (i.e., perceived stress, irritation, and screening positive for depression, anxiety, comorbidity, and psychological distress). We evaluated differences in mental health by sex and academic level. Overall, although self-rated health did not differ by sex and academic level, females and lower academic level were associated with less favorable mental health. Males reported higher prevalence of high self-esteem, and higher engagement (all *p* ≤ 0.04). Conversely, mean perceived stress and cognitive/emotional irritation were higher among females, as were rates for positive screenings for anxiety, anxiety and depression comorbidity, and psychological distress (*p* < 0.001 for all). Likewise, lower academic level (BSc) was associated with lower rates of high self-esteem (*p* ≤ 0.001), increased perceived stress (*p* < 0.001), and higher prevalence of positive screening for depression, anxiety, comorbidity, and psychological distress (*p* ≤ 0.002 for all), while higher academic level (PhD) was linked to increased student engagement (*p* < 0.001 for all). Although the effect sizes of sex and academic level on student mental health were modest, these findings support a need for action to establish and expand early detection and prevention programs, on-campus advisory services, and peer counseling that focus on the sex-specific and academic-study-level-specific factors, as well as mental health and career development resources for students. Academics and policy makers need to consider multipronged intervention strategies to boost confidence of students and their academic career.

## 1. Introduction

There is an increased risk of mental illness during early adulthood [1], and many psychiatric conditions have an onset at 17–24 years of age [2]. Hence, the mental health of university students is a public health concern [3], as it can be negatively influenced by the numerous stressors encountered during the college years and increase the risk of multiple physical and psychiatric illnesses [4]. Student stressors encompass, among others, new living environments, increased responsibilities, and being away from the family home. Furthermore, students are subject to stressors including the need to achieve academic success in the face of financial constraints, adapting to changes in academic workloads, and the psychosocial adjustments in one’s social and support networks [5,6].

Currently, the largest study on stress among students in Germany, based on a survey of 18,000 students, concluded that 53% were excessively stressed due to time constraints, pressure to perform, or fear of being overwhelmed. Thus, the prevalence of high levels of perceived stress was higher among students compared to the working general population (50%) [7]. Likewise, students at seven UK universities exhibited high perceived stress levels and depression scores [8], and undergraduates in Egypt reported various mental health complaints (e.g., nervousness, anxiety, depressive mood, mood swings, fear, difficulty to concentrate) [9]. Furthermore, depressive symptoms affected one-third of medical students [10], and were highly prevalent among freshmen in Poland, Bulgaria, and Germany (23–39%) [11], while prevalence rates were higher in sophomores (37%) compared to freshmen (27%) in China [12]. Of concern, 40% of undergraduates in the USA were severely depressed [13]. In this regard, the concept of “irritation” has demonstrated increased attraction in studies on work-related mental health. Introduced by Mohr et al. [14] and applied to the university student setting [15], irritation describes a state of psychological exhaustion that cannot be assuaged by rest. Moreover, previous findings suggest that irritation might mediate the effect of stressors on the development of depression [16]. Thus, irritation is less than a mental illness but more than mere fatigue, and can result in more serious impairments in the longer term [15].

In addition, recent studies highlight the emerging trends towards a positive psychology, which does not only focus on mental illness, but also on the positive aspects of mental health. These cover personal strengths and resources (e.g., self-esteem, engagement), which are linked to resiliency, intrinsic motivation, and better academic performance [17]. For instance, 84–90% of college students in India, Iran, and Spain reported at least medium self-esteem [18,19,20], for which negative correlations with student irritation [21] and independent associations with symptoms of depression, anxiety, and stress [20] have been demonstrated. Similarly, engagement has also been shown to be inversely associated with burnout [22] and depressive symptoms [12] among university students.

Differences pertaining to sex and academic level of study also exist, although findings remain inconsistent. In terms of sex, depressive symptoms were higher among female college students [23]. In agreement, in the UK, female students exhibited higher perceived stress and depressive symptoms [8]; in Turkey, females at four universities had higher depression levels [24]; and in Germany, more female students reported a mental health problem [25]. Conversely, there were no sex differences in self-esteem among Chinese college students [26]. As for academic level, no association between self-esteem and years of study were found at six colleges in Saudi Arabia [27]. There is some evidence that grades accomplished in 10th grade predicted self-esteem in the 12^th^ grade [28], and others observed that despite an inconsistency between school performance and self-esteem, the two do have a positive relationship [29].

The consequences of mental health symptoms are numerous. They negatively affect academic achievement, forecasting lower grade point average and college dropout; stress-related health problems are common among university students, where academic stress increased disordered eating among female university students and was associated with lower self-esteem, and in the USA, >10% of undergraduates had seriously thought about suicide [11,13,30,31,32,33,34]. The beliefs and feelings students have about themselves (self-esteem) are key determinants of academic success [35]. Likewise, among undergraduates in the UK and Egypt, the frequency of many health impairments worsened with the increase in perceived stress [8,9]. Unsurprisingly, the mental health stressors that college students face is a public health concern [3], university students’ mental health needs continue to grow [36,37,38], and a “campus mental health crisis” exists due to the increased frequency and magnitude of problems among university students [34,39].

However, the literature reveals knowledge gaps. Few studies investigated the triad of college students’ mental health, sex, and level of academic study. Usually, studies examined one or two of these components or narrowly focused on one or two mental health domains, e.g., only stress, only depression, stress and depression, only self-esteem, etc. [8,9,11,19,24,27]. While such an approach is useful, it neglects other domains that are necessary to provide the broader picture. Furthermore, most studies had modest sample sizes, thus limiting their generalizability [18,19,27]. The present study bridges these knowledge gaps by extensively describing multiple mental health domains as a function of gender and academic levels comprising Bachelor of Science (BSc), Master of Science (MSc), and Doctoral (PhD) students. The specific objectives were to evaluate both the positive aspects of mental health (i.e., high self-rated health, self-esteem, and student engagement) as well as the unfavorable aspects of mental health (i.e., perceived stress, irritation, and symptoms of depression, anxiety, depression and anxiety comorbidity, and psychological distress), analyzed by sex and academic level of study.

## 2. Materials and Methods

### 2.1. Setting

As part of a student health project, we conducted a cross-sectional online survey at one of the largest universities in Germany (Westfälische Wilhelms-Universität, WWU Münster). The University of Münster has 15 departments with almost 45,000 students, of which 56% are female, and 7% are foreign students. We used the EvaSys evaluation software (version 8.0, Electric Paper Evaluationssysteme GmbH, Lüneburg, Germany), a web-based tool allowing for adaptive questioning and plausibility checks. The focus of the survey was to describe the health behavior of the students [40] and to assess various mental health indicators in order to identify starting points for data-driven student health promotion interventions. The questionnaire was provided in validated German and English language versions. The University Ethics Committee approved the study (2019-07-TU), which was conducted during the 2019 summer term (pre-pandemic). We approached all regularly enrolled students (*n* = 42,630) by email, inviting them to participate. Therefore, no a priori sample size calculation was performed. The mailing informed potential participants about the survey objectives and time required for completion (20–30 min), as well as voluntariness, anonymity, and privacy. Embedded code numbers ensured that each student could only participate once. In case of non-participation, students received a maximum of two reminder emails at intervals of five days. All participants provided informed consent before completing the questionnaire. Regarding students’ academic level, we only evaluated data of BSc, MSc, and PhD students because other degrees (diploma, state exam) do not allow for a differentiation of undergraduate and graduate status.

### 2.2. Variables

#### 2.2.1. Subjective Health

Self-rated health is the subjective evaluation of one’s overall health status and plays an integral role in psychological research and general population surveys [41]. It is correlated with objective health indicators and shows positive correlations with measures of subjective well-being [42]. We assessed subjective health using a single question: “In general, how would you rate your health?/Wie würden Sie Ihren Gesundheitszustand im Allgemeinen beschreiben?”. The item was rated on a 5-point Likert scale ranging from 1 = ‘very bad’ to 5 = ‘very good’, representing an economic, valid, and reliable umbrella indicator of the general health status [43].

#### 2.2.2. Self-Esteem

Self-esteem is vital for understanding one’s well-being and success and is defined as the sense of self-worth and self-respect, thus reflecting positive and negative attitudes towards the self [44,45]. A single-item scale has been developed to measure global self-esteem as an efficient alternative to the established Rosenberg Self-Esteem Scale covering ten items [46]. We assessed self-esteem using a single question: “In general, I have high self-esteem”/“Im Allgemeinen habe ich ein hohes Selbstwertgefühl” using the anchors 1 = ‘not very true of me’ to 5 = ‘very true of me’ [46]. The single-item self-esteem scale has been demonstrated to be a valid, reliable, and economical instrument for measuring global self-esteem [45].

#### 2.2.3. Student Engagement (UWES-9-SF)

To follow up on emerging trends towards a positive psychology that focuses on student strengths and optimal functioning rather than on weaknesses and malfunctioning, we additionally assessed student engagement, defined as an active and positive state of mind that is characterized by vigor, dedication, and absorption [17,47,48]. Derived from the Utrecht work engagement scale (UWES), the validated student form (UWES-9-SF) covers nine items (three items for its vigor, dedication, and absorption subscales) [17,49,50]. UWES-9-SF uses a 7-point Likert scale with the anchors 0 = ‘never’ to 6 = ‘always’, with higher mean scores indicating higher student engagement. Internal consistency for the total scale was excellent (α = 0.92), and good for the subscales ranging between α = 0.82–0.84, which is comparable to a recent study among German university students [50].

#### 2.2.4. Perceived Stress Scale (PSS)

Stress is considered an important contributing factor to physical and psychological ill health, comprising cardiovascular diseases [51,52] or depression and anxiety [53]. The Perceived Stress Scale (PSS) is a well-established instrument to assess the degree the respondents experienced life as unpredictable, uncontrollable, and overloaded within the past month [54,55]. The PSS is a reliable and economic measure available in a validated German language version [54]. Ten items were rated on a 5-point Likert scale ranging from 0 = ‘never’ to 4 = ‘very often’. The analysis of the PSS requires recoding the items 4, 5, 7, and 8. A total score ranging from 0–40 can be calculated by summing the score of all ten items with higher scores indicating elevated levels of perceived stress. Reliability analyses revealed good internal consistency (Cronbach’s alpha: α = 0.88).

#### 2.2.5. Student Irritation Scale

The construct “irritation” was introduced by Mohr et al. in 1986 for its application in occupational contexts [14], and later adapted to university students by Hiemisch et al. in 2018 with a validated German language version [15]. Irritation describes a state of exhaustion that can no longer be relieved by rest and is considered a valid indicator for the presence of mental strain and a precursor of mental ill health [15]. Two facets are subsumed under the label irritation, namely cognitive irritation (or rumination), describing a behavior of not being able to switch off from work/studies, associated with reinforced goal orientation, and emotional irritation (or irritability), describing a more aggressive behavior for which it is considered a more severe symptomatology, linked to goal defense/abandonment [15,56]. Both subscales comprise three items rated on a 7-point Likert scale, ranging from 1 = ‘strongly disagree’ to 7 = ‘strongly agree’ [14], and higher values indicate elevated irritation. Internal consistencies (total scale α = 0.82, cognitive irritation α = 0.84, emotional irritation α = 0.86) were comparable to a previous study examining the reliability and validity of the student irritation scale [15].

#### 2.2.6. Patient Health Questionnaire for Depression and Anxiety (PHQ-4)

Depression and anxiety represent common mental disorders associated with disability, which is even greater when they co-occur. The Patient Health Questionnaire for Depression and Anxiety (PHQ-4) is derived from the well-established measures PHQ-9 and Generalized Anxiety Disorder 7 (GAD-7) used to assess depressive and anxiety disorders, respectively. The PHQ-4 combines both measures using two two-item scales, the PHQ-2, and the GAD-2, consisting of core criteria for depression and anxiety. Overall, previous studies support the reliability and validity of the PHQ-4 and its subscales as ultra-brief screening tools for depression and anxiety in the general population [57,58]. Both tools use a four-point Likert scale ranging from 0 = ‘not at all’ to 3 = ‘nearly every day’, resulting in a subscale sum score of 0 to 6, and a PHQ-4 composite score ranging from 0 to 12. Scores ≥ 3 on both subscales indicate positive screenings for major depressive disorder (83% sensitivity and 92% specificity) [57] and generalized anxiety disorder (86% sensitivity and 83% specificity) [59]. Additionally, scores ≥ 3 on both screens are indicative of anxiety and depression comorbidity [57], and PHQ-4 composite scores ≥ 6 are “yellow flags” signaling the presence of a depressive or an anxiety disorder, for which the general term “psychological distress” is used in this paper [58]. Reliability analyses revealed good internal consistency for the total scale (α = 0.84), and acceptable to good internal consistency for the subscales PHQ-2 (α = 0.76) and GAD-2 (α = 0.80), respectively.

### 2.3. Statistical Analysis

We implemented ipsative mean imputation in cases where not more than a single item of the total scale of the PSS or the subscales of the UWES-9-SF and student irritation scale were missing [60]. Subjects with two or more missing items were excluded from the analyses. Likewise, we performed listwise deletions for subjects with any missing values in the PHQ-4 due to the brevity of the scale [60]. As a measure of reliability, Cronbach’s alpha determined the internal consistencies of the scales used in the current survey. We present categorical variables using frequencies (percentages) and continuous variables using means (±standard deviation). Chi-square independence tests and multivariate analyses of variance (MANOVA) evaluated the samples for any sex differences across the categorical and continuous variables, respectively. For categorical variables, differences based on the academic degree pursued were analyzed by generalized linear models (GLM) using binary logistic regression analyses adjusted for sex. Continuous variables were analyzed using multivariate analyses of covariance (MANCOVA) with sex as the only covariate. As a measure of the magnitude of effects of sex and academic level on student mental health, we calculated odds ratios (OR) with 95% confidence intervals (CI_95%_) and partial eta squared (*η*_p_^2^) for categorical and continuous variables, respectively. All analyses were performed using IBM SPSS v.28 (IBM Corporation, Armonk, NY, USA), and significance level was set at *p* < 0.05.

## 3. Results

A total of 4244 students participated in the survey, corresponding to a response rate of 10%. Regarding the objectives of this study, data of 3353 (67% female) students were analyzed. The remaining data were acquired from students who were enrolled in state examination or diploma degree programs, in which no differentiation between undergraduate and graduate studies is possible. Students from all 15 departments were represented in the data, with participation rates ranging between 7–22%, whereby many students are usually enrolled in multiple departments, e.g., students in teaching degree programs are enrolled in educational studies.

### 3.1. Student Mental Health by Sex

Most students (70%) reported good to very good subjective health and high self-esteem (60%). Conversely, 32% and 34% screened positive for depression and anxiety, respectively, while the depression–anxiety comorbidity was reported in 22% of the sample. Table 1 shows that females had lower odds of high self-esteem, whereas female sex was associated with higher odds of a positive screening for anxiety, comorbidity, and psychological distress. Furthermore, Table 2 shows that female sex was associated with higher perceived stress as well as cognitive and emotional irritation, although the effect of sex on negative mental health aspects was small (*η*_p_^2^ = 0.01–0.02). On the contrary, despite significant sex differences, no effects of sex on positive mental health aspects were found (*η*_p_^2^ ≤ 0.01).

### 3.2. Student Mental Health by Academic Level of Study at University

Generally, although no link between academic level and self-rated health was found (*χ*^2^ = 4.140, *df* = 2, *p* = 0.126), the academic level pursued was positively associated with the favorable domains of student mental health and inversely associated with its negative aspects. Table 3 shows that BSc students had significantly lower odds of high self-esteem compared to PhD students. Consistently, BSc students had significantly higher odds of positive screenings on depression, anxiety, depression–anxiety comorbidity, and psychological distress. 

Significant group differences were found regarding the positive and negative aspects of mental health (Table 4). Student engagement and its subscales were significantly lower among BSC and MSC students compared to PhD students (all *p* < 0.001), while perceived stress and emotional irritation were the only scales in which negative aspects of mental health were significantly higher among BSc students compared to PhD students. Again, the effect of academic level on positive and negative mental health aspects was small (*η*_p_^2^ = 0.01–0.02). 

## 4. Discussion

Mental disorders are increasingly recognized as leading causes of disease burden [61]. Unsurprisingly, university students’ well-being and mental health is a public health concern. Hence, researchers and campus stakeholders are assessing the mental health of students, as well as associated risk factors in terms of student characteristics [62]. The current study appraised a range of mental health domains among under- and postgraduate students at a large university in Germany, focusing on sex and academic level. Our main findings were that 70% of students rated their health as at least good with no sex differences. Male sex was associated with high self-esteem and student engagement. Conversely, females were associated with higher levels of perceived stress, cognitive and emotional irritation, as well as higher odds for positive screenings for anxiety, depression and anxiety comorbidity, and psychological distress. As for academic level, lower levels of study were associated with lower odds for high self-esteem, higher odds for positive screenings for depression, anxiety, comorbidity, and psychological distress, as well as with higher perceived stress and emotional irritation. In principle, effect sizes of sex and academic level were modest (*η*_p_^2^ = 0.01–0.02). Below we discuss each mental health domain vis-a-vis sex and academic study level.

### 4.1. Student Mental Health by Sex

Overall, 70% of our students reported good to very good subjective health with no sex or academic level differences. The literature is equivocal regarding student self-rated health, since our findings are 15% lower compared to a previous survey in three European countries, in which 9% and 36% rated their health as excellent and very good, respectively [11], and 10% lower compared to a recent study among French college students [63]. Conversely, only 40% of Australian university students rated their health as very good to excellent [64], and the present findings are comparable with Egypt [65], with the exception that, compared to males, female students rated their health more commonly as poor [65]. Of note, despite poorer self-rated health among female students, paradoxically more men were represented in the poorest health category [66]. Given that poor subjective health is associated with health literacy and the tendency to have insufficient information to manage their health or navigate the healthcare system, focusing on students with low self-rated health might be a promising first step for campus prevention programs [65].

Almost 60% of participants in the present study reported high self-esteem, reflecting that >40% had low self-esteem, higher than the 16%, 13%, and 10% low self-esteem rates reported among college students in Iran, India, and Spain, respectively [18,19,20], most likely due to the use of different assessment instruments. Nevertheless, the relationships between sex and self-esteem seem inconsistent. Among Chinese college students, no sex differences were observed [26], while in Iran, more males (19%) than females (15%) reported low self-esteem, contrasting with our findings. Given that self-esteem, or the sense of self-worth and self-respect, has close relationships with the given society’s cultural norms [67], comparisons of student self-esteem in Germany vs. non-Western societies, e.g., Iran, India, or China, with different cultural, religious, and social perspectives, might not be completely justified. Certainly, self-esteem is a multi-dimensional construct, its levels may not always be fully explained by individual factors, and the range of tools that appraise self-esteem generally or as domain-specific measures [68] renders comparisons even more complex.

As for student engagement, our results align well with previous studies among German and Spanish university students [50,69]. Although we found higher overall engagement among males, absorption was the only subscale for which significant sex differences were observed, contrary to a recent study among Australian students, where only dedication was associated with sex [70]. Frequently, there are specific reasons for premature termination of studies, with performance problems (35% _males_, 25% _females_) followed by lack of motivation (20% _females_) and the desire for more practical work (16% _males_) [71]. Notably, although males reported higher engagement suggesting higher motivation and performance that should stimulate well-being, recent reports showed that one in three university students in Germany dropped out of their studies, with an 8% higher proportion among males [71].

In terms of perceived stress, our findings are in line with the largest national survey on stress (18,000 students) in Germany that reported a mean value of 19.8 on Cohen’s PSS: 53% of the sample reported high levels of stress, and females had significantly higher perceived stress compared to their male counterparts [7]. Likewise, females generally exhibited higher perceived stress across seven universities in the UK [8], in the USA [72], or in Saudi Arabia [73]. Although a stressful life is not a sufficient cause for pathology or mental illness, high perceived stress levels might interfere with the motivation for productivity and academic performance, and even prove harmful when exceeding the individual’s capability to cope [73]. Several reasons may explain the sex differences observed, including a more stressful perception of otherwise equal challenging or adverse events among females, or a lower propensity to express emotions as probable indicators of weakness and low masculinity among males [73]. Expectedly, overall, cognitive and emotional irritation were significantly higher among females compared to males, highlighting findings from a previous study of presenting standardization data for the original instrument used in the occupational context [74]. With irritation being a state of psychological strain resulting from goal discrepancy including rumination, in the sense of reinforced goal-achievement efforts (cognitive irritation), and irritability, in the sense of goal-defense tendencies (emotional irritation) [56], the student form of the irritation scale might prove useful to plan interventions in the university context before study dropouts or even psychological illnesses occur.

Across university students from 23 countries, the prevalence of depressive symptoms was 19% _male_ and 22% _female_ [23], lower than our current 29% _male_ and 33% _female_. In the present study, although more females reported depression, the differences were not significant, supporting a meta-analysis of depression amongst students where females were more likely to be depressed, but differences were not statistically significant [10]. Nevertheless, across college students in the UK, Finland, Nepal, and Turkey, females were significantly more likely to have depressive symptoms [8,24,75,76]. A recent overview of systematic reviews of depression amongst students found that sex was a prominent factor associated with depressive symptoms across 51 primary studies [77]. Likewise, among students in three European countries, female sex was associated with higher depression scores in bivariate analyses and after adjusting for other variables in the multivariate analysis, but when perceived stress was included in the model, sex differences disappeared [11].

In terms of anxiety, 34% of the current sample screened positive, less than in the USA where 61% of college students reported feeling overwhelming anxiety [13]. We observed that female sex was associated with a higher prevalence of screening positive for anxiety, supporting previous research among college/university students from China, the USA, and Germany [78,79,80]. However, among undergraduates in India, there was a significant association of male sex with anxiety only among second year medical students [81], and others reported no significant gender differences in anxiety [82,83]. Overall, these results suggest that policy makers need to pay attention to mental health disorders, since recent findings indicate that 85% of students diagnosed with a mental health disorder prematurely drop out of their studies [84].

### 4.2. Student Mental Health by Academic Level of Study at University

We found that academic level was positively, yet not significantly, associated with rates of good and very good subjective health, generally concurring with previous reports from Italy and Brazil [85,86]. Likewise, lower academic level was significantly associated with fewer students reporting high self-esteem, which also supports previous findings [28,29,87], while others observed significant differences between academic levels on only one out of seven items assessing self-esteem [27]. Generally, we observed that academic level was positively associated with engagement, suggesting that as academic levels of study progress, students report increased vigor (or higher levels of energy and resilience), are more absorbed in their studies, and have a more intense level of dedication to academic tasks. Our scores align well with recent research among Chilean university students [88], but were considerably higher compared to reports from Spain [22]. Whilst we agree with others that dedication is the factor where students obtained higher scores [69,88], our findings contrast with others, where students in the first year exhibited higher levels in the three subscales of student engagement [69]. Engaged students are characterized by high levels of energy and mental resilience while studying (vigor), by being strongly involved their studies and experiencing a sense of significance, enthusiasm, or inspiration (dedication), and by being focused while finding it difficult to detach from one’s studies (absorption) [88]. 

In this regard, Porru et al. [85] showed that student life challenges, e.g., faculty shortcomings (including passive learning, lack of opportunities to influence the curriculum, or insufficient preparation for the future profession), an unsupportive climate, and high workloads were associated with mental and self-rated health and could be addressed by adopting innovative teaching methods with more participatory roles of students in their education process, which might impact student engagement and motivation [89,90], improve self-esteem, and lower levels of perceived stress/resilience [91,92].

We observed an inverse association between academic level and perceived stress, confirming the largest national survey using Cohen’s PSS among German students, where BSc students had a 101.3% mean stress level compared to the whole sample, while MSc and PhD students had mean values of 99.2% and 95.6% respectively [7]. Likewise, the current study observed that significantly more BSc than MSc and PhD students screened positive for depression, and that there was a decline in depressive symptoms with progression in academic level from BSc to PhD (34%, 29%, and 26%, respectively). We also found an inverse association between anxiety and increasing academic level. Probably, these findings might result from facing new challenges with the beginning of university studies, encompassing new living environments, increased responsibilities, and being away from home while being subjected to financial constraints and changes in academic workloads [5,6]. With progress in academic levels, these factors seem to have less of an impact as students become more familiar with the university setting and have more financial resources at their disposal, as is usually the case for PhD students [10]. Typically, 50% of students report psychological distress, as opposed to 11% in the general population, and 30% indicate that chronic stress affects their academic performance [84]. Furthermore, stress is associated with unfavorable health behavior and leads to about 35-50% of university students who drop out prematurely due to insufficient coping skills under chronic stress [84]. All these factors point to the need of stress prevention and stress management programs in the university setting.

### 4.3. Limitations

This study has limitations. Being a descriptive cross-sectional prevalence study, no causal inferences regarding the direction of effects can be drawn. Likewise, conclusions drawn from a convenience sample are limited in significance, and the response rate of 10% must also be considered a limitation in terms of selection bias. Although international students make up a significant percentage of students at the university (7%), we did not specifically ask to differentiate between foreign and non-foreign students. Therefore, although interesting in principle, we cannot draw conclusions about mental health differences between German and international students. Lastly, no sociodemographic characteristics as potential influencing factors on student mental health have been considered in the current analyses. The strengths of the present study are that its underlying research questions, i.e., student mental health as a function of sex and academic level, were analyzed by implementing a multitopic survey across various endpoints, including positive and negative aspects of mental health, in a large and representative sample (*N* > 3350).

## 5. Conclusions

The present findings need be interpreted as a call for action. Both academics and policy makers need to establish and/or expand mental health resources for the entire student body, with particular focus on graduate students, and to consider appropriate intervention strategies to tackle the high prevalence of student psychological burden. Such efforts should focus on the structural and behavioral prevention levels and might encompass, among others, reviewing academic and exam schedules, appropriate communication and interaction with faculty staff, student peer counseling, or easily accessible programs promoting stress management skills and resiliency training specifically targeting the student body as part of a mandatory university curriculum, where applicable. To promote the implementation of sustainable and purposeful health promoting measures, universities should plan and assess their strategies based on periodic health reports.

## Figures and Tables

**Table 1:** Student mental health by sex for categorical variables.

Mental Health	Total	Females	Males	*χ* ^2^	OR [95% CI]	*p*-Value
Positive aspects						
(Very) good subjective health	2325 (69.7)	1546 (69.3)	779(70.6)	0.53	0.94 [0.81; 1.10]	0.465
High self-esteem	1984 (59.5)	1300 (58.3)	684 (62.0)	4.23	0.86 [0.74; 0.99]	0.040
Negative aspects						
Positive screen for						
Depression	1021 (31.5)	705 (32.5)	316 (29.4)	3.16	1.16 [0.99; 1.35]	0.076
Anxiety	1109 (34.2)	827 (38.1)	282 (26.2)	44.47	1.73 [1.47; 2.03]	<0.001
Comorbidity	712 (21.9)	521 (24.0)	191 (17.8)	16.19	1.46 [1.22; 1.76]	<0.001
Psychological distress	923 (28.4)	670 (30.9)	253 (23.5)	18.86	1.45 [1.23; 1.72]	<0.001

Values represent *n* (%) and *p*-values are based on Chi-square independence tests (two-sided); OR = odds ratios; CI = confidence interval.

**Table 2:** Student mental health by sex for continuous variables.

Mental Health	Total	Females	Males	*F*	*η*_p_^2^	Mean Difference [95% CI]	*p*-Value
Positive aspects							
Student engagement	3.27 ± 1.13	3.23 ± 1.12	3.35 ± 1.16	7.45	<0.01	−0.12 [−020; −0.03]	0.006
Vigor	2.89 ± 1.14	2.88 ± 1.12	2.91 ± 1.19	0.64	<0.01	−0.03 [−0.12; 0.05]	0.423
Dedication	3.64 ± 1.32	3.61 ± 1.31	3.70 ± 1.36	3.59	<0.01	-0.09 [−0.19; 0]	0.058
Absorption	3.29 ± 1.28	3.22 ± 1.27	3.43 ± 1.31	20.91	0.01	−0.22 [−0.31; −0.13]	<0.001
Negative aspects							
Perceived stress	19.17 ± 7.08	19.91 ± 6.86	17.72 ± 7.27	70.39	0.02	2.19 [1.68; 2.70]	<0.001
Irritation	23.59 ± 7.98	24.30 ± 7.81	22.16 ± 8.18	51.90	0.02	2.13 [1.55; 2.72]	<0.001
Cognitive irritation	13.33 ± 4.99	13.77 ± 4.85	12.44 ± 5.14	51.90	0.02	1.33 [0.97; 1.69]	<0.001
Emotional irritation	10.26 ± 4.64	10.53 ± 4.60	9.71 ± 4.68	21.75	0.01	0.81 [0.47; 1.14]	<0.001

Values represent mean ± standard deviation; mean difference (females vs. males) and *p*-values are based on MANOVA; CI = confidence interval.

**Table 3:** Student mental health by academic level for categorical variables.

Mental Health	BSc	MSc	PhD	*χ* ^2^	*p*-Value	OR [95% CI]		
						BSc	MSc	PhD
Positive aspects								
(Very) good Subjective health	1341 (68.4)	669 (71.1)	315 (72.7)	4.14	0.126	0.82 [0.65; 1.03]	0.93 [0.72; 1.20]	1
High self-esteem	1098 (56.0)	594 (63.1)	292 (67.7)	25.71	<0.001	0.62 [0.49; 0.77]	0.83 [0.65; 1.05]	1
Negative aspects								
Depression	646 (33.8)	265 (28.9)	110 (26.3)	12.23	0.002	1.41 [1.11; 1.79]	1.13 [0.87; 1.46]	1
Anxiety	720 (37.7)	280 (29.5)	109 (26.0)	24.78	<0.001	1.63 [1.28; 2.07]	1.18 [0.91; 1.53]	1
Comorbidity	463 (24.2)	181 (19.8)	68 (16.2)	14.68	<0.001	1.59 [1.20; 2.10]	1.22 [0.90; 1.66]	1
Psychological distress	596 (31.2)	235 (25.7)	92 (22.0)	17.33	<0.001	1.55 [1.21; 2.00]	1.18 [0.89; 1.55]	1

Values represent *N* (%) and *p*-values are based on binary logistic regression analyses adjusted for sex; OR = odds ratio; CI = confidence interval.

**Table 4:** Student mental health by academic level for continuous variables.

Mental Health	BSc	MSc	PhD	*F*	*p*-Value	*η*_p_^2^	Mean Difference [95% CI]		
							BSc	MSc	PhD
Positive aspects									
Student engagement	3.20 ± 1.11	3.25±1.17	3.63 ± 1.09	24.07	<0.001	0.02	−0.42 [−0.57; −0.27]	−0.38 [−0.54; −0.22]	0
Vigor	2.83 ± 1.11	2.89±1.19	3.14 ± 1.16	16.48	<0.001	0.01	−0.31 −0.46; −0.16]	−0.26 [−0.42; −0.10]	0
Dedication	3.59 ± 1.32	3.59±1.32	3.97 ± 1.28	14.65	<0.001	0.01	−0.37 [−0.54; −0.20]	-0.38 [−0.56; −0.19]	0
Absorption	3.19 ± 1.26	3.27±1.33	3.78 ± 1.17	35.94	<0.001	0.02	-0.58 [−0.74; −0.42]	-0.49 [−0.67; −0.32]	0
Negative aspects									
Perceived stress	19.87 ± 6.86	18.41 ± 7.23	17.67 ± 7.30	22.03	<0.001	0.01	1.94 [1.04; 2.84]	0.47 [−0.51; 1.45]	0
Irritation	23.77 ± 7.86	23.61 ± 8.18	22.75 ± 8.15	1.58	0.206	<0.01	0.76 [−0.27; 1.79]	0.59 [−0.53; 1.72]	0
Cognitive irritation	13.30 ± 4.87	13.51 ± 5.16	13.08 ± 5.15	0.64	0.525	<0.01	0.06 [−0.58; 0.70]	0.27 [−0.44; 0.97]	0
Emotional irritation	10.47 ± 4.62	10.10 ± 4.61	9.67 ± 4.71	4.97	0.007	<0.01	0.71 [0.11; 1.30]	0.33 [−0.32; 0.98]	0

Values represent mean ± standard deviation; mean differences (BSc vs. PhD and MSc vs. PhD) and *p*-values are based on MANCOVA adjusted for sex; CI = confidence interval.

## Data Availability

The data presented in this study are available on reasonable request from the first author (C.M.).
